# Survival Risk Prediction Models of Gliomas Based on IDH and 1p/19q

**DOI:** 10.7150/jca.43805

**Published:** 2020-04-27

**Authors:** Han Zou, Chang Li, Siyi Wanggou, Xuejun Li

**Affiliations:** 1 Xiangya School of Medicine, Central South University, 172 Tongzipo Road, Changsha, Hunan 410013, China; 2 Department of Neurosurgery, Xiangya Hospital, Central South University, 87 Xiangya Road, Changsha, Hunan 410008, China; 3 Hunan International Scientific and Technological Cooperation Base of Brain Tumor Research, Xiangya Hospital, Central South University, No. 87, Xiangya Road, Changsha, Hunan 410008, China

**Keywords:** survival analysis, prediction model, ncRNA, molecular features, glioma.

## Abstract

Gliomas have been classified into different molecular subtypes based on their molecular features. To explore the prognostic factors of different subtypes of gliomas, we performed a univariate survival analysis based on the RNA-seq data of 653 patients obtained from The Cancer Genome Atlas. We identified 12205 (20.18%), 6125 (10.13%) and 5206 (8.61%) genes associated with the overall survival (OS) of the IDH-wildtype, IDH-mutation 1p/19q intact and IDH-mutation 1p/19q codeletion gliomas, respectively. Pathway enrichment analysis revealed that OS related genes were mainly involved in alcoholism, systemic lupus erythematosus, hematopoietic cell lineage and diabetes. The OS related genes were further selected using Lasso regression, and three prognostic risk score models were constructed to effectively predict the OS of the patients with different subtypes of gliomas. In total, 76 signature genes were identified and were selected to construct the three models. Moreover, neither of the 76 genes overlapped between different models, which suggested the enormous difference among the three subtypes, although some signature genes (SERPINA5, RP11.229A12.2 and RP11.62F24.2) were also identified as the OS related genes in different glioma subtypes. Interestingly, five genes (RP11.229A12.2, RP11.62F24.2, C3orf67, RP11.275H4.1 and TBX3) played opposing roles (protective or risk factor) in different subtypes. Additionally, the prognosis models consisted of a substantial proportion of non-coding RNA (58.74%, 70.13% and 58.11% in the IDH-wildtype, IDH-mutation 1p/19q intact and IDH-mutation 1p/19q codeletion). Furthermore, multivariate analysis integrating clinical variables demonstrated that risk group predicted by the prognostic models was an independent prognostic factor for gliomas. In conclusion, we have constructed and validated three models that have the potential to predict the prognosis of glioma patients. The genes and pathways identified in this study require further investigation for their underlying mechanisms and potential clinical significance in improving the OS of the glioma patients.

## Introduction

Glioma is the most common malignant Central Nervous System (CNS) tumor, with a 5-year overall survival (OS) no greater than 35% 1. Despite multiple therapy options, it is still challenging for researchers and doctors to improve the OS 2-5. The reasons are complex but may lie in RNAs because gene expression profiling has been verified as a promising tool to classify tumors and predict the prognosis of cancer 6, 7. To find out the OS related genes, gliomas were classified into several molecular subtypes because not only their prognosis differs stupendously between each other, but also the molecular alteration is an origin that causes glial or precursor cells evolving into different histological types 8, 9.

The World Health Organization has reclassified glioma according to the IDH and 1p/19q status in 2016 10. In this study, we selected the genes associated with OS to establish three prognostic models by lasso regression to predict the survival risk of patients from The Cancer Genome Atlas (TCGA) with IDH-wildtype glioma, IDH-mutation 1p/19q intact gliomas and IDH-mutation 1p/19q codeletion gliomas, respectively. All of the three models are able to accurately predict the survival rate of 1 year, 3 years and 5 years. Therefore, the genes in these models are the potential factors that can influence the OS greatly. Also, these genes will be able to give insight into glioma and provide directions for subsequent research.

## Materials and Methods

### Data collection

All data of the 702 patients including RNA-seq data, clinical information and molecular features were obtained from TCGA (https://cancergenome.nih.gov/). The data collection was conducted in compliance with the publication guidelines and policies for the protection of human subjects provided by TCGA. Ensembl gene ID annotated RNA expression profiles were based on the V24 (hg19) of GENCODE (https://www.gencodegenes.org). Patients with the following conditions were eliminated in the study: (1) no IDH or 1p/19q molecular features (2) no survival information.

### Identification of OS related genes

The analysis was carried out among 233 IDH-wildtype, 254 IDH-mutation 1p/19q intact and 166 IDH-mutation 1p/19q codeletion glioma samples using the “survival” and “survminer” packages in R/Bioconductor. All the total 60483 genes were analyzed one by one to perform a univariate survival analysis, and genes with *P*-value < 0.05 were selected for model establishment.

### Lasso regression

Because genes affect each other can cause several colinear gene groups, we performed Lasso using the “lars” and “glmnet” packages in R/Bioconductor to reduce the collinearity influence and enhance the prediction accuracy and interpretability of the statistical mode. Cross-validation was used to select the regularization parameter. In order to quantify the risk of OS, a standard form of risk score (RS) for each patient was calculated combining the expression levels of the RNAs (Expi) and LASSO coefficients (Li), Risk score = 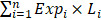.

### ROC and Kaplan-Meier curve

To investigate the performance of the prognostic classifier in predicting patient outcome, the area under the curve (AUC) of the receiver-operator characteristic (ROC) was calculated and compared. To validate the efficiency and repeatability of the models, we randomly divided the patients in each group into training set and validation set with a ratio of 6:4 by R. To divide the patients into the high or low risk group in the training set, the best cutoff was determined with the “survival” and “survminer” packages in R/Bioconductor. Kaplan-Meier curves were plotted to estimate the survival status for patients in the high risk and the low risk group in both the training set and the testing set. In order to further assess the accurate role of the genes, heatmap was drawn using the R/Bioconductor package “ggplot2”.

### Cox regression

Whether the prognostic value of the multi-RNA-based classifier is independent of clinical features was assessed by multivariate Cox regression model.

All the statistical tests were done with R software version 3.5.1 and corresponding fundamental package. The figures were drawn with R software with or without subsequent modification by Adobe Illustrator CS6.

### Enrichment analysis of KEGG pathways

Genes associated with OS were included in the Kyoto Encyclopedia of Genes and Genomes (KEGG) pathway enrichment analysis using the WEB-based Gene SeT AnaLysis Toolkit (http://www.webgestalt.org). The hypergeometric test statistical method and the Benjamini-Hochberg multiple test adjustment method were used. All genes from human beings were used as reference. Top 10 pathways with at least 10 genes involved were considered significantly enriched (FDR < 0.05).

## Results

**Figure 1:**
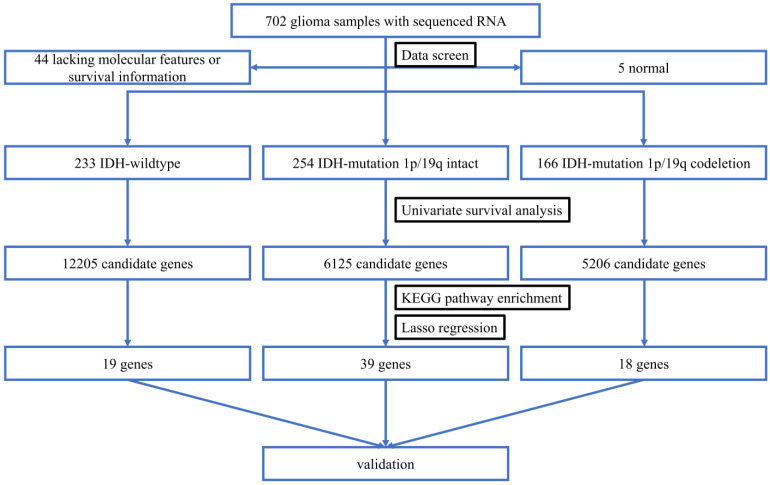
The overall workflow chart. 702 glioma samples with RNA-seq data were obtained from TCGA. After data screening, 49 samples were excluded. The remaining 653 samples were divided into three subtypes. Univariate survival analysis identified 12205, 6126, and 5206 candidate genes that were associated with the OS of IDH-wildtype, IDH-mutation 1p/19q intact and IDH-mutation 1p/19q codeletion gliomas, respectively. These genes were included in the KEGG pathway enrichment analysis. Three models were established using Lasso regression based on the OS-related genes. The models were validated in the testing sets.

The overall flowchart was summarized in Figure 1.

### Clinicopathological features of patients in the TCGA

The clinicopathological features of 233 IDH-wildtype, 254 IDH-mutation 1p/19q intact and 166 IDH-mutation 1p/19q codeletion patients were listed in Table 1. The features of IDH-wildtype gliomas are quite different from that of the IDH-mutation gliomas, while the two types of IDH-mutation are similar to each other. Consistent with previous studies, IDH-wildtype patients were older than IDH-mutation patients at diagnosis (50-70 years old vs 20-50 years old), with a poorer prognosis and mainly consisted of glioblastoma and G4 instead of G2 and G3. IDH-mutation 1p/19q intact and IDH-mutation 1p/19q codeletion patients had no significant difference among age, grade, OS and gender, except the histologic types because IDH-mutation 1p/19q intact patients mainly harbored astrocytoma while IDH-mutation 1p/19q codeletion patients mainly harbored oligodendroglioma.

### Identification of genes associated with OS

**Figure 2:**
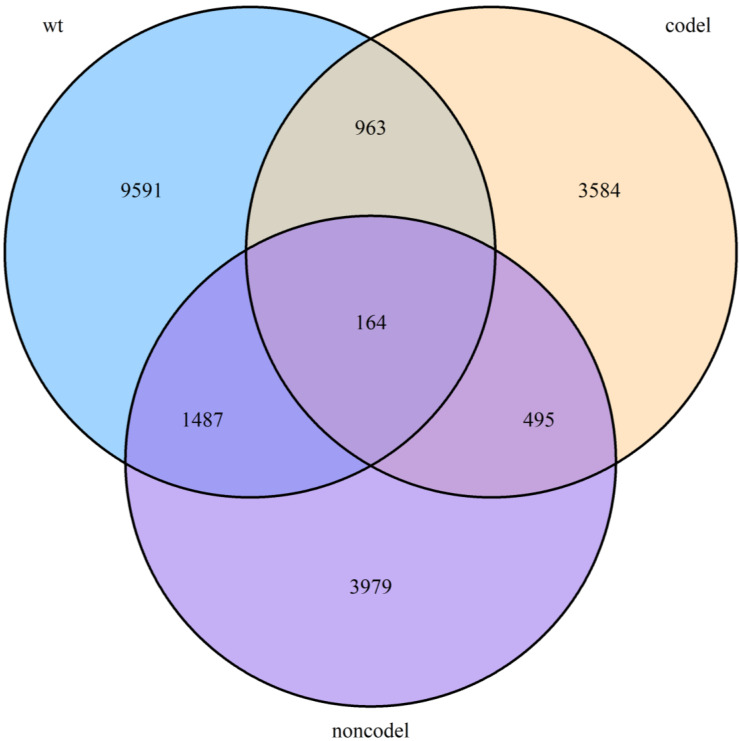
Overlapping condition of the three gene sets identified by univariate survival analysis. A Venn diagram presenting the overlapping condition of genes related to OS selected by univariate survival analysis. wt: IDH-wildtype; noncodel: IDH-mutation 1p/19q intact; codel: IDH-mutation 1p/19q codeletion.

To begin with, we identified three sets of genes by performing univariate survival analysis using Cox Proportional Hazard Regression Model, with the threshold of *P*-value set as 0.05. The overlapping condition of the genes contained in the three sets is shown in Figure 2. Respectively, 12205 (20.18%), 6125 (10.13%) and 5206 (8.61%) genes were included in the IDH-wildtype, IDH-mutation 1p/19q intact and IDH-mutation 1p/19q codeletion set. The non-coding RNAs accounted for 58.74%, 70.13% and 58.11% of the OS related genes in the IDH-wildtype, IDH-mutation 1p/19q intact and IDH-mutation 1p/19q codeletion set respectively.

### Further genes screening and model establishment

**Figure 3:**
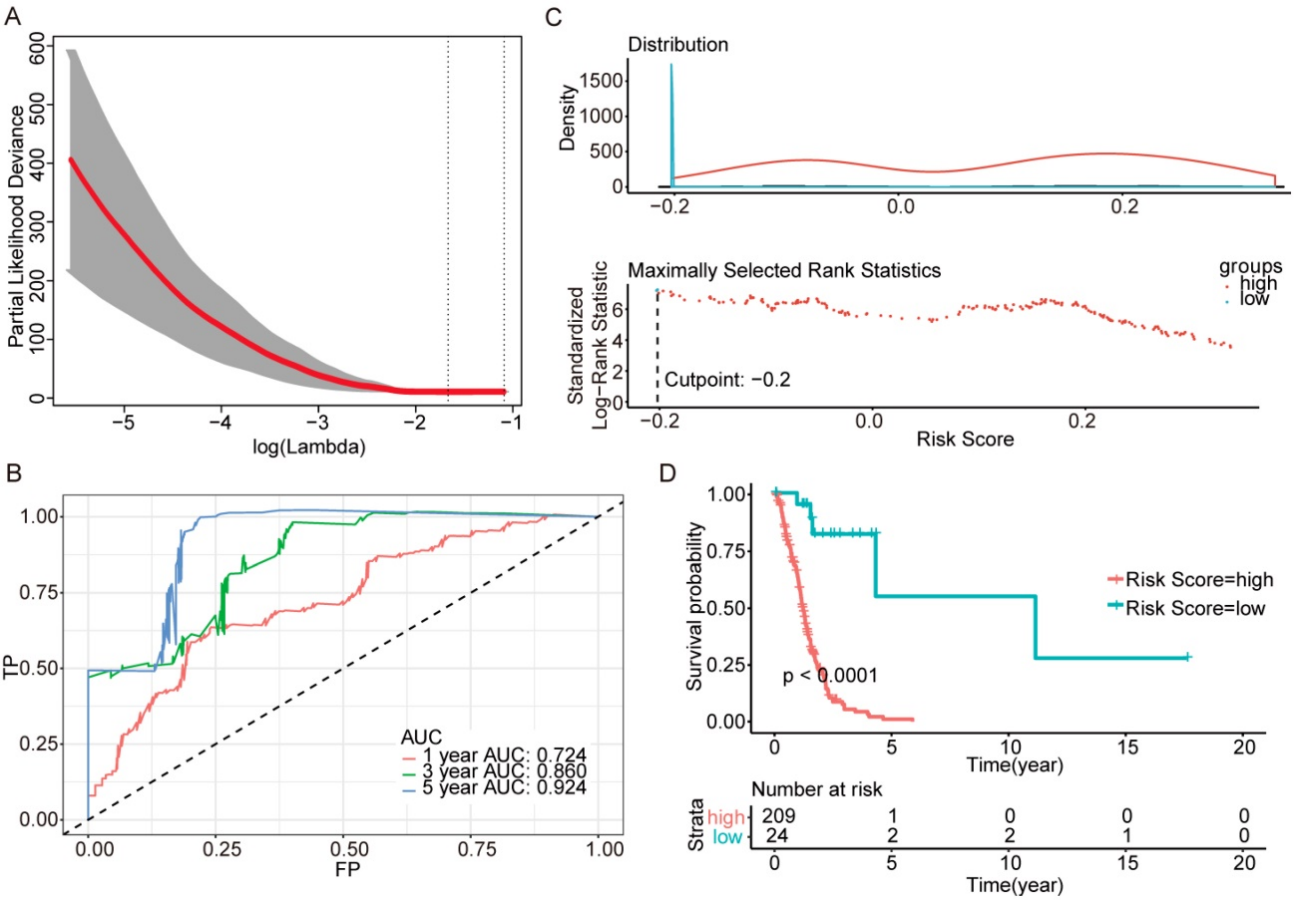
Further genes screening and model establishment of IDH-wildtype. (A) Cross-validation of Lasso regression. (B) ROC curve and AUC of the model. (C) Cutpoint determined with R/Bioconductor packages “survival” and “survminer”. (D) Kaplan-Meier curve of high risk and low risk groups.

**Figure 4:**
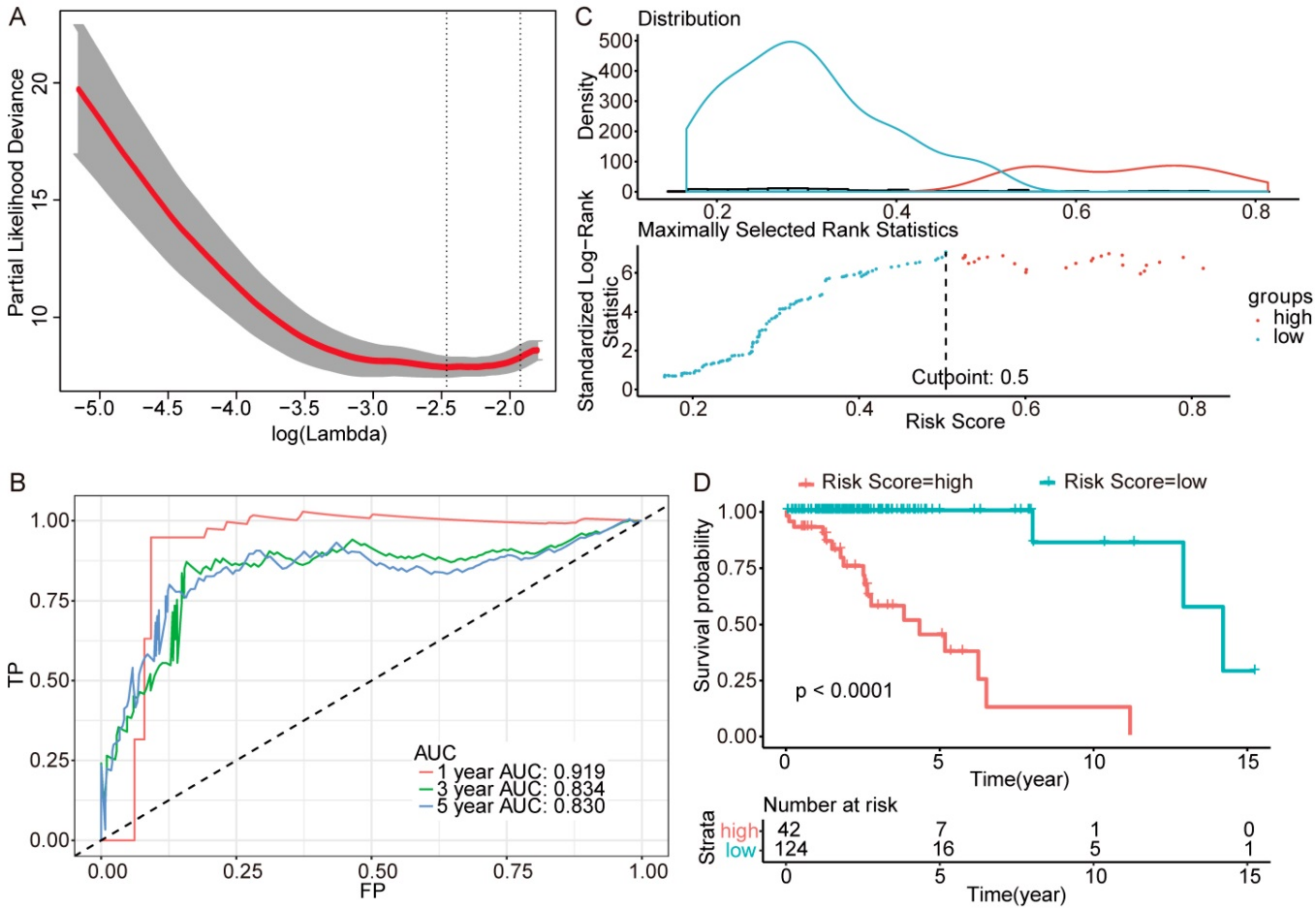
Further genes screening and model establishment of IDH-mutation 1p/19q codeletion type. (A) Cross-validation of Lasso regression. (B) ROC curve and AUC of the model. (C) Cutpoint determined with R/Bioconductor packages “survival” and “survminer”. (D) Kaplan-Meier curve of high risk and low risk groups.

**Figure 5:**
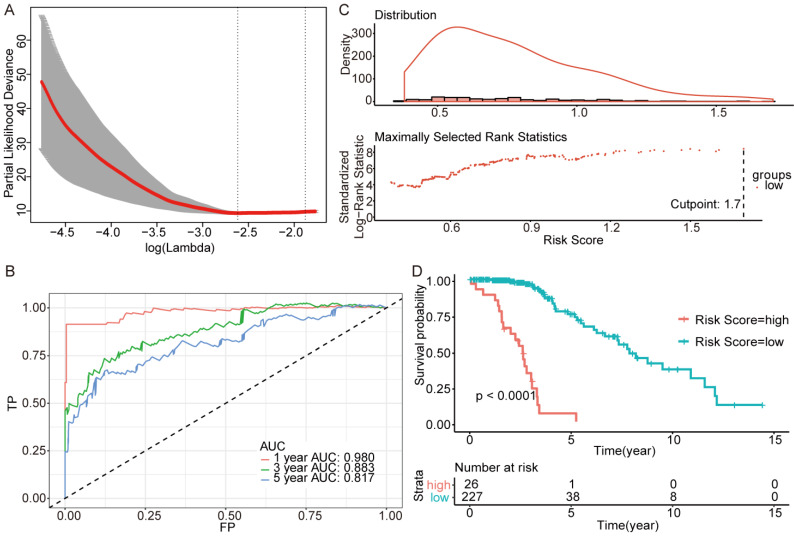
Further genes screening and model establishment of IDH-mutation 1p/19q intact type. (A) Cross-validation of Lasso regression. (B) ROC curve and AUC of the model. (C) Cutpoint determined with R/Bioconductor packages “survival” and “survminer”. (D) Kaplan-Meier curve of high risk and low risk groups.

We next screened genes associated with OS based on Lasso. Three sets of significant genes optimally predicting the survival risk score of patients with glioma were selected with cross-validation method (Figure 3A, Figure 4A and Figure 5A) and three survival risk score systems based on the 76 genes were constructed (Table 2). Moreover, non-coding RNAs accounted for almost half in IDH-mutation types while less in IDH-wildtype.

Then we plotted time-dependent ROC curves for 1, 3 and 5 years. the AUCs were high enough to support the efficiency of the genes and models (IDH wildtype 1-year AUC: 0.724, 3-year AUC: 0.860, 5-year AUC: 0.924; IDH mutation 1p/19q codeletion 1-year AUC: 0.919, 3-year AUC: 0.834, 5-year AUC: 0.830; IDH mutation 1p/19q intact 1-year AUC: 0.980, 3-year AUC: 0.883, 5-year AUC: 0.817) (Figure 3B, Figure 4B and Figure 5B).

To investigate the relationship between RS and survival status of each group of the glioma patients, Kaplan-Meier analysis and log-rank test were conducted. The optimal cutoff was calculated with the methods described above and shown in Figure 3C, Figure 4C and Figure 5C. Obviously, patients with higher RS generally had a significantly worse OS than those with lower RS (all *P*-values of the three groups are less than 0.0001) (Figure 3D, Figure 4D and Figure 5D). Furthermore, the ratios of high-risk patients and low-risk patients are 8.7, 0.3 and 0.1 in IDH wildtype, IDH mutation 1p/19q codeletion and IDH mutation 1p/19q intact patients respectively. The results corresponded to the recognition that prognosis of IDH mutation is much better than that of IDH wildtype.

### Validation of the models in the TCGA

**Figure 6:**
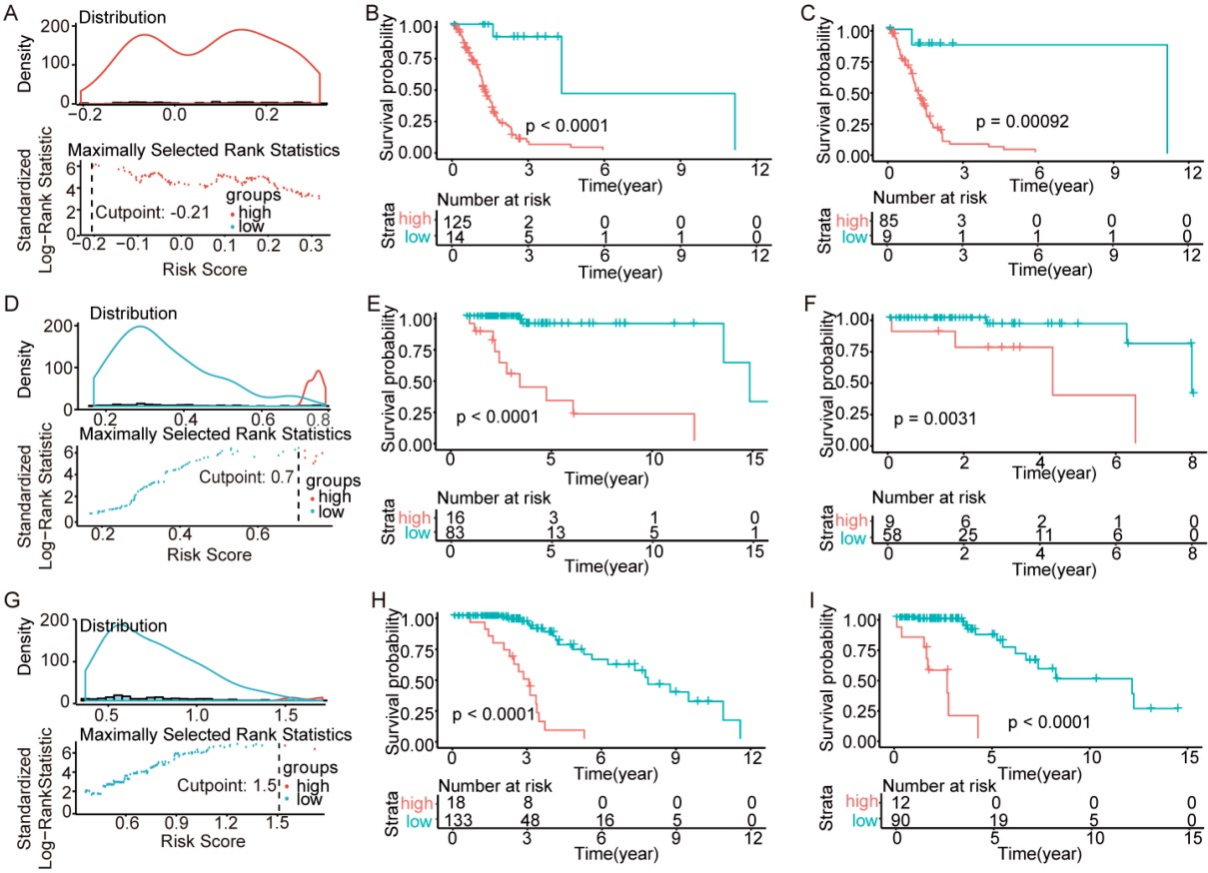
Validation of the models in the TCGA. We randomly divided the patients into training sets and testing sets by 6:4. (A-C) IDH-wildtype. (D-F) IDH-mutation 1p/19q codeletion type. (G-H) IDH-mutation 1p/19q intact type. (A), (D) and (G) RS cutoff of the training sets. (B), (E) and (H) Kaplan-Meier curve of the training sets. (C), (F) and (I) Kaplan-Meier curve of the testing sets.

To test the models, we randomly divided all patients into training sets and testing sets at a ratio of 6:4. Within the training sets, RS cutoff (Figure 6A, Figure 6D and Figure 6G), Kaplan-Meier analysis and log-rank test were made for each subtype. Obviously, patients with higher RS generally had a significantly worse OS than those with lower RS (p<0.0001; Figure ​6B, Figure 6E and Figure 6H). Additionally, the RS cutoff was calculated in the training set, and Kaplan-Meier analysis was performed for both the training and testing sets to evaluate whether the models were powerful enough to separate patients into high-risk group and low-risk group. Consequently, all the subtypes' models were good enough to separate high-risk group and low-risk group apart from each other in the testing set (Figure 6C, Figure 6F and Figure 6I).

### Gene function inversion in different subtypes

**Figure 7:**
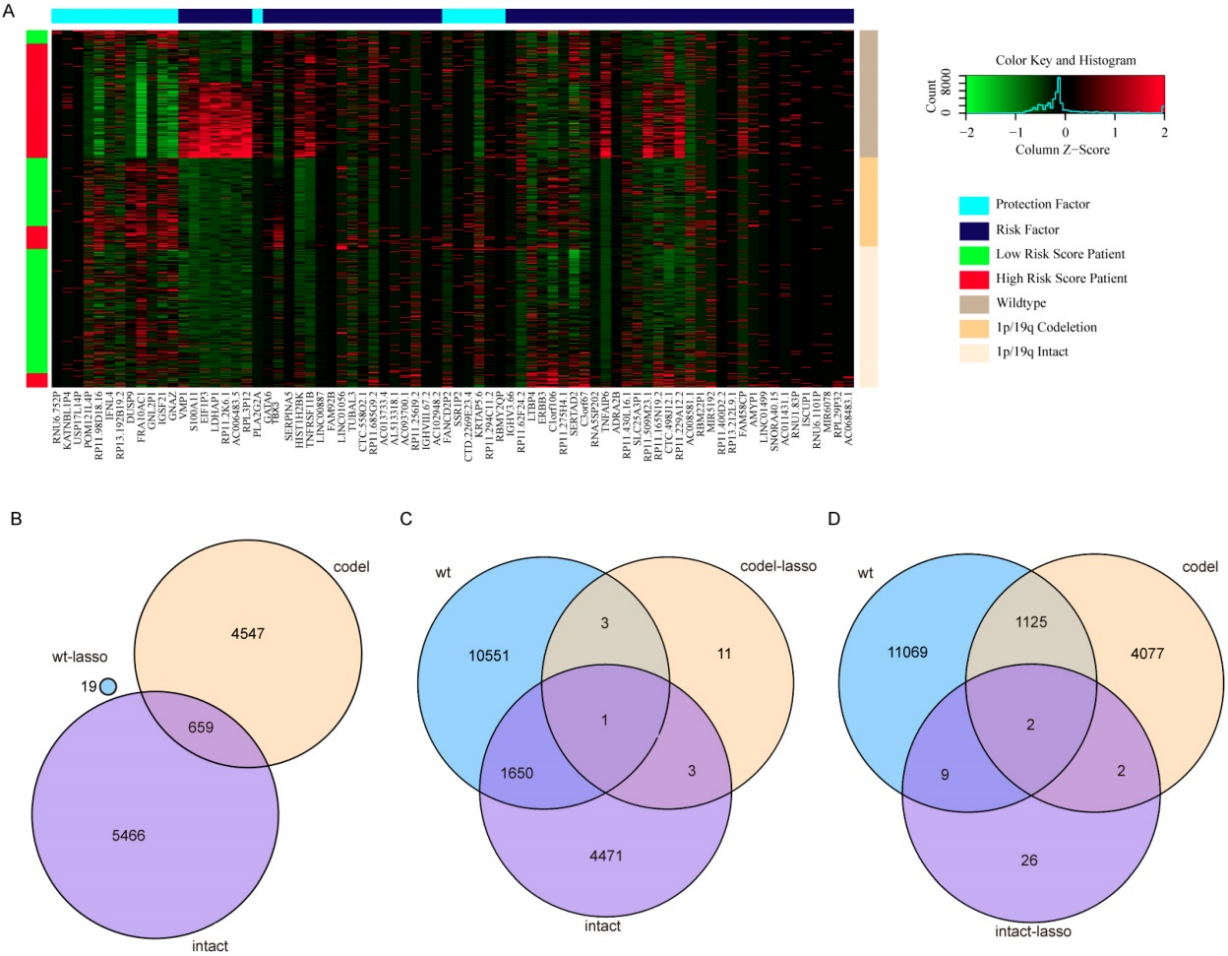
Heatmap and overlapping condition in different subtypes. (A) Heatmap of the 76 genes selected in the three risk score models. (B) The overlapping condition among genes in the prognostic model of IDH-wildtype and genes related to OS of the other two subtypes. (C) The overlapping condition among genes in the prognostic model of IDH-mutation 1p/19q codeletion type and genes related to OS of the other two subtypes. (D) The overlapping condition among genes in the prognostic model of IDH-mutation 1p/19q intact type and genes related to OS of the other two subtypes.

**Figure 8:**
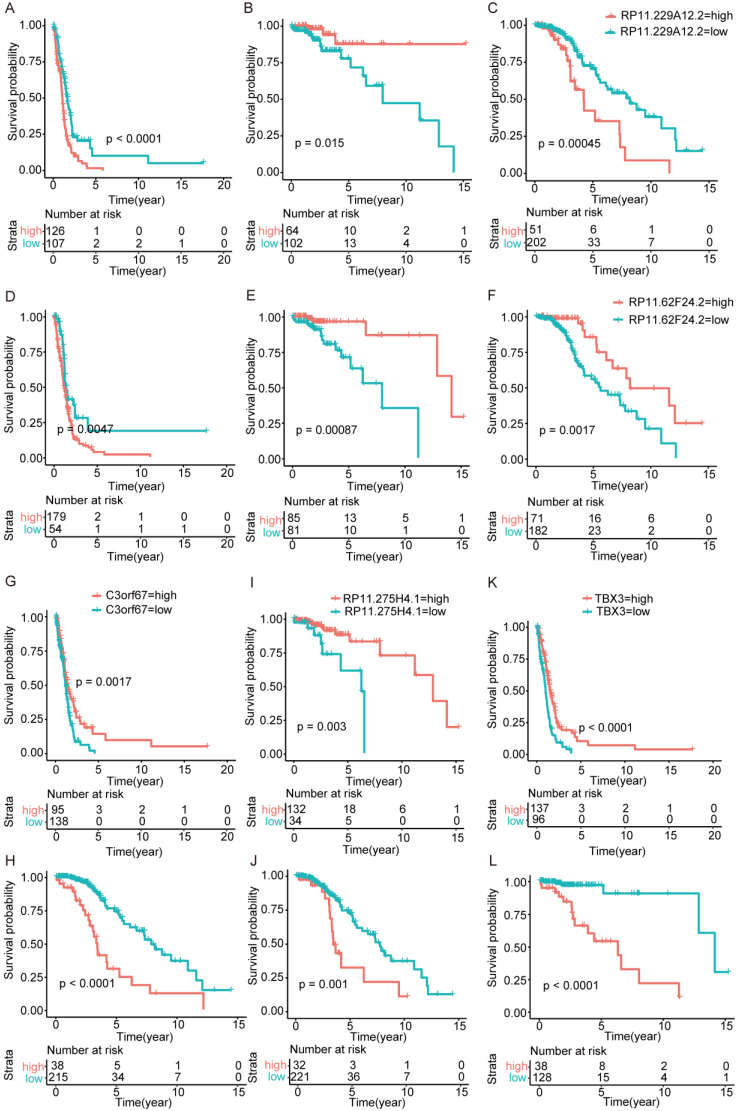
Kaplan-Meier curves of genes played opposing roles in different subtypes. (A-C) RP11.229A12.2 expression in IDH-wildtype, IDH-mutation 1p/19q codeletion type and IDH-mutation 1p/19q intact type. (D-F) RP11.62F24.2 expression in IDH-wildtype, IDH-mutation 1p/19q codeletion type and IDH-mutation 1p/19q intact type. (G-H) C3orf67 expression in IDH-wildtype and IDH-mutation 1p/19q intact type. (I-J) RP11.275H4.1 expression in IDH-mutation 1p/19q codeletion type and IDH-mutation 1p/19q intact type. (K-L) TBX3 expression in IDH-wildtype and IDH-mutation 1p/19q codeletion type.

Surprisingly, there are no overlapping genes between different models, which suggested the enormous difference among the three types. The overlapping condition among genes in model of one subtype and genes related to OS of the other two subtypes was shown in Figure 7. SERPINA5 in IDH-mutation 1p/19q codeletion type, RP11.229A12.2 and RP11.62F24.2 in IDH-mutation 1p/19q intact type were also identified as OS related genes of other two subtypes. Additionally, the heatmap also showed that a protective or risk factor of a glioma subtype can sometimes play a different or even an opposing role in other subtypes of gliomas. Genes with reverse function (changing from protective factor to risk factor or from risk factor to protection factor) included RP11.229A12.2, RP11.62F24.2, C3orf67, RP11.275H4.1 and TBX3. The Kaplan-Meier curves were plotted for the five genes in Figure 8.

### Prognostic value of the models is independent of clinical features

To assess whether the models represent an independent indicator in glioma patients, the effect of each clinicopathologic feature on survival was analyzed by Cox regression. As shown in Table 3, in the univariate analysis, gender was not a powerful factor in all the three subtypes; histology was not powerful in IDH-mutation types but powerful in IDH-wildtype; age was not a powerful factor in 1p/19q intact type but powerful in the others. Both grade and risk score were powerful in all the three subtypes. Furthermore, hazard ratio (HR) of risk score was the highest (IDH wildtype 33.72 (14.13-80.45), *P*-value = 2.20E-15, IDH-mutation 1p/19q codeletion 18.07 (6.928-47.13), *P*-value = 3.28E-9 and IDH-mutation 1p/19q intact 10.59 (6.319-17.74), *P*-value < 2E-16).

After multivariable adjustment, the risk scores of these models remained powerful and independent. Except for age in IDH-wildtype and 1p/19q codeletion types which remained powerful, other factors, even grade was not identified as powerful or independent. The HRs of risk score calculated by the three models (IDH wildtype 125.7044 (32.1569-491.390), *P*-value = 3.67E-12, IDH-mutation 1p/19q codeletion 7.590 (2.4850-23.181), *P*-value = 0.00037 and IDH-mutation 1p/19q intact 10.6033 (6.1490-18.284), *P*-value < 2E-16) were also much higher than other factors, which illustrates the efficiency of the three models.

### KEGG pathway enrichment analysis

To identify the key pathways associated with OS, we performed KEGG pathway enrichment analysis for the three sets of OS related genes (Table 4). Cell-energy related pathways, GTP and cAMP were included. This may illustrate that energy requirement of cancer cells was related to the OS. Surprisingly, the genes of IDH-mutation were enriched in alcoholism, systemic lupus erythematosus, hematopoietic cell lineage and diabetes. This might give a cue of the connection among these diseases and might represent the relationship between unhealthy lifestyle and the OS of glioma patients.

## Discussions

In this study, we identified 76 genes to establish three risk-score models that can effectively predict the overall survival of patients with IDH-wildtype, IDH-mutation 1p/19q intact and IDH-mutation 1p/19q codeletion gliomas respectively.

The genes identified by univariate survival analysis are enriched in alcoholism, systemic lupus erythematosus and hematopoietic cell lineage. Recent studies suggested that alcoholism may share a common pathway with glioma through proteasome and oxidative stress, and disulfiram (a drug used for the treatment of alcoholism for decades) has been proven effective in glioma treatment 11, 12. As for the systemic lupus erythematosus, more studies are needed to investigate its relationship with glioma 13. IDH mutation can promote leukemogenesis, and hematopoietic cell lineage was suggested in gliomas in previous studies 14-17. However, whether glioma patient with these diseases has a short OS and the underlying mechanisms require further studies. Additionally, the RAGE pathway in diabetes and diabetes itself and insulin usage are proved associated with glioma 18, 19.

Further studies are required for the 76 genes in the models to explore their roles in affecting the OS of patients with glioma, as only a small part of them has been reported to be related to the OS of cancer. Additionally, the non-coding RNA accounted for almost half of the 76 genes but has seldom been studied in glioma, and they should be attached enough attention for their increasing importance in glioma. Some non-coding RNAs have been verified to play roles in glioma 20-22, and therapies targeting non-coding RNAs have become promising in glioma 23, 24. Molecular pathways regulating the expression of protective RNAs or risk RNAs might provide potential therapeutic targets to improve the prognosis of glioma patients.

Interestingly, several genes changed from protective factors to risk factors in different glioma types and vice versa. For example, TBX3 is associated with a number of cancers, including head and neck squamous cell carcinoma, gastric, breast, ovary, cervical, pancreatic, bladder, liver cancers and melanoma 25. Its special stemness function and role as a microenvironment-dependent and epigenetically regulated lineage-commitment factor makes it a risk factor in cancers 25, 26. Besides, it has been related to the hormonal therapy resistance in breast cancers 27. In this study, TBX3 is a protective factor in IDH-wildtype but a risk factor in IDH-mutation 1p/19q codeletion gliomas. These opposing roles may suggest a complex underlying mechanism for the pathway regulation in gliomas 28. Although we have associated these genes with different molecular features of gliomas, such as IDH and 1p/19q, further studies are needed to explore this phenomenon.

One limitation of this study is that we did not validate the models in other databases because no studies with qualified data for a reliable validation were available. With the rapid progress of the next-sequencing applied in cancer, the robustness of the models would be further validated in the future.

## Figures and Tables

**Table 1:** Clinicopathologic features for the glioma patients

Characteristics	Group		
Age (years)	IDH- wildtype	IDH-mutation 1p/19q intact	IDH-mutation 1p/19q codeletion
1~10	0	0	0
11~20	0	7	2
21~30	13	55	13
31~40	13	84	45
41~50	30	48	32
51~60	66	19	37
61~70	64	9	15
71~80	31	1	6
81~90	8	0	0
91~100	0	0	0
Histology			
oligodendroglioma	19	37	116
oligoastrocytoma	15	67	30
astrocytoma	52	110	4
glioblastoma	139	9	0
Grade			
G2	19	114	80
G3	67	100	70
G4	139	9	0
Gender			
Male	137	130	83
Female	88	93	67
Vital Status			
Alive	68	206	145
Dead	165	48	21
Days to Death (mean±SD)	466±570	1068±957	1003±976
Median Days to Death	382	836	710.5

**Table 2:** The coefficient of three models

wt	coefficient	co	coefficient	intact	coefficient
AC006483.5	8.18E-03	AC013733.4	2.15E-01	AC008581.1	5.64E-02
DUSP9	-2.22E-04	AC093700.1	2.45E-01	AC011431.1	3.54E-01
EIF1P3	1.37E-03	AC102948.2	8.42E-01	AC068483.1	1.67E+00
FRA10AC1	-4.40E-05	AL513318.1	2.32E-01	ADRA2B	2.04E-03
GNAZ	-4.99E-06	CTC.558O2.1	7.48E-02	AMYP1	1.96E-01
GNL2P1	-1.85E-05	FAM92B	1.84E-02	C1orf106	6.99E-05
IFNL4	-1.89E-03	GATA6	5.41E-06	C3orf67	3.94E-04
IGSF21	-9.39E-06	HIST1H2BK	3.17E-03	CTC.498J12.1	2.40E-02
KATNBL1P4	-1.34E-01	IGHVIII.67.2	4.92E-01	CTD.2269E23.4	-3.01E-02
LDHAP1	2.66E-03	LINC00887	1.44E-02	ERBB3	3.15E-05
POM121L4P	-9.14E-03	LINC01056	2.16E-02	FAM58CP	1.74E-01
RNU6.752P	-2.03E-01	PLA2G2A	-3.29E-05	FANCD2P2	5.35E-02
RP11.2K6.1	3.62E-03	RP11.256I9.2	4.02E-01	IGHV3.66	-1.12E-03
RP11.98D18.16	-1.94E-03	RP11.685G9.2	1.08E-01	ISCUP1	6.39E-01
RP13.192B19.2	-1.87E-03	SERPINA5	5.11E-04	KRTAP5.6	-2.52E-02
RPL3P12	1.90E-02	TBX3	8.05E-05	LINC01499	2.60E-01
S100A11	4.45E-06	TNFRSF11B	7.82E-03	LTBP4	6.87E-06
USP17L14P	-9.91E-03	TUBAL3	2.42E-02	MIR5192	1.10E-01
VMP1	2.14E-06			MIR6078	9.37E-01
				RBM22P1	6.22E-02
				RBMY2QP	-4.14E-03
				RNA5SP202	8.46E-04
				RNU1.83P	5.04E-01
				RNU6.1101P	7.79E-01
				RP11.165N19.2	1.01E-02
				RP11.229A12.2	5.62E-02
				RP11.275H4.1	8.32E-05
				RP11.294C11.2	-8.09E-03
				RP11.400D2.2	1.13E-01
				RP11.430L16.1	2.11E-03
				RP11.509M23.1	3.62E-03
				RP11.62F24.2	-1.03E-03
				RP13.212L9.1	1.17E-01
				RPL29P32	1.02E+00
				SERTAD2	2.35E-04
				SLC25A3P1	3.50E-03
				SNORA40.15	2.72E-01
				SSR1P2	-2.00E-01
				TNFAIP6	1.02E-03

wt: IDH-wildtype; co: IDH-mutation 1p/19q codeletion; int: IDH-mutation 1p/19q intact.

**Table 3:** Univariate and multivariate analysis of clinicopathologic features and risk score.

Variables	Categories	Univariate analysis			Multivariate analysis			
		HR (95% CI)	coef	*P*-value (Wald test)	HR (95% CI)	coef	*P*-value (Wald test)	*P*-value of multivariate analysis
wt								
Age	age < 50, 0; age > 50, 1	1.033 (1.02-1.047)	0.032541	7.93E-07	1.0155 (1.0001-1.031)	0.01535	0.0488	<2e-16
Gender	male, 1; female, 0	1.277 (0.9176-1.778)	0.2447	0.147				
Grade	I, 1; II, 2; III, 3; IV, 4	2.153 (1.596-2.904)	0.7668	5.18E-07	0.6956 (0.3873-1.249)	-0.36295	0.2244	
Histology	oligodendroglioma, 1; oligoastrocytoma, 2; astrocytoma, 3; glioblastoma, 4	1.596 (1.302-1.955)	0.4672	6.48E-06	0.7680 (0.5406-1.091)	-0.26393	0.1407	
Risk score		33.72 (14.13-80.45)	3.518	2.20E-15	125.7044 (32.1569-491.390)	4.83393	3.67E-12	
co								
Age	age < 50, 0; age > 50, 1	1.139 (1.081-1.2)	0.13001	1.04E-06	1.106 (1.0457-1.170)	0.10076	0.000428	8e-09
Gender	male, 1; female, 0	0.799 (0.3307-1.931)	-0.2243	0.618				
Grade	I, 1; II, 2; III, 3; IV, 4	6.748 (2.132-21.36)	1.9093	0.00116	2.264 (0.5529-9.272)	0.81722	0.255885	
Histology	oligodendroglioma, 1; oligoastrocytoma, 2; astrocytoma, 3; glioblastoma, 4	1.026 (0.3287-3.201)	0.02531	0.965				
Risk score		18.07 (6.928-47.13)	2.8942	3.28E-09	7.590 (2.4850-23.181)	2.02681	0.000374	
intact								
Age	age < 50, 0; age > 50, 1	1.015 (0.9891-1.042)	0.01509	0.257				<2e-16
Gender	male, 1; female, 0	1.134 (0.6331-2.032)	0.1259	0.672				
Grade	I, 1; II, 2; III, 3; IV, 4	2.187 (1.382-3.459)	0.7824	0.000825	0.9961 (0.6002-1.653)	-0.003885	0.988	
Histology	oligodendroglioma, 1; oligoastrocytoma, 2; astrocytoma, 3; glioblastoma, 4	1.301 (0.8963-1.887)	0.2629	0.166				
Risk score		10.59 (6.319-17.74)	2.3598	<2e-16	10.6033 (6.1490-18.284)	2.361161	<2e-16	

wt: IDH-wildtype; co: IDH-mutation 1p/19q codeletion; intact: IDH-mutation 1p/19q intact.

**Table 4:** KEGG pathway enrichment

Types	Description	Number	FDR
wt	positive regulation of GTPase activity	226	1.54E-09
wt	regulation of GTPase activity	240	2.30E-09
wt	positive regulation of cellular component organization	369	6.57E-09
wt	positive regulation of hydrolase activity	301	6.57E-09
wt	positive regulation of molecular function	547	6.57E-09
wt	regulation of hydrolase activity	409	1.09E-08
wt	positive regulation of catalytic activity	465	1.19E-07
wt	establishment of localization in cell	582	1.56E-07
wt	vesicle-mediated transport	426	2.35E-06
wt	intracellular transport	491	3.19E-06
int	Alcoholism	54	0
int	Systemic lupus erythematosus	46	0
int	Transcriptional misregulation in cancer	33	0.002252
co	ECM-receptor interaction	27	2.93E-05
co	HIF-1 signaling pathway	24	0.04264
co	Alcoholism	35	0.04264
co	Systemic lupus erythematosus	28	0.04264
co	Hematopoietic cell lineage	22	0.04264
co	Cholinergic synapse	24	0.04264
co	cAMP signaling pathway	37	0.04264
co	AGE-RAGE signaling pathway in diabetic complications	22	0.04264
co	Proteoglycans in cancer	37	0.04264
co	Focal adhesion	37	0.04264

wt: IDH-wildtype; int: IDH-mutation 1p/19q intact; co: IDH-mutation 1p/19q codeletion; FDR: false discovery rate.

## Abbreviations

CNS: Central Nervous System
OS: overall survival
TCGA: The Cancer Genome Atlas
KEGG: Kyoto Encyclopedia of Genes and Genomes
RS: risk score
AUC: area under the curve
ROC: receiver-operator characteristic
HR: hazard ratio
